# Do Health Professionals Need Additional Competencies for Stratified Cancer Prevention Based on Genetic Risk Profiling?

**DOI:** 10.3390/jpm5020191

**Published:** 2015-06-09

**Authors:** Susmita Chowdhury, Lidewij Henneman, Tom Dent, Alison Hall, Alice Burton, Paul Pharoah, Nora Pashayan, Hilary Burton

**Affiliations:** 1 PHG Foundation, 2 Worts Causeway, Cambridge CB1 8RN, UK; E-Mails: tom.dent@phgfoundation.org (T.D.); alison.hall@phgfoundation.org (A.H.); hilary.burton@phgfoundation.org (H.B.); 2 Department of Clinical Genetics, Section Community Genetics, and EMGO Institute for Health and Care Research, VU University Medical Center, Amsterdam, PO Box 7057, 1007 MB, The Netherlands; E-Mail: L.Henneman@vumc.nl; 3 UCL Division of Infection and Immunity, University College London, Cruciform Building, 90 Gower Street, London WC1E 6BT, UK; E-Mail: alice.burton.14@ucl.ac.uk; 4 Departments of Oncology and of Public Health and Primary Care, University of Cambridge, Cambridge CB1 8RN, UK; E-Mail: pp10001@medschl.cam.ac.uk; 5 UCL Department of Applied Health Research, University College London, 1-19 Torrington Place, London WC1E 6BT, UK; E-Mail: n.pashayan@ucl.ac.uk

**Keywords:** genetic risk, risk-assessment, common-disease prevention, competence, health professionals, risk-stratified prevention, risk-stratified screening, knowledge, skills, risk-tools

## Abstract

There is growing evidence that inclusion of genetic information about known common susceptibility variants may enable population risk-stratification and personalized prevention for common diseases including cancer. This would require the inclusion of genetic testing as an integral part of individual risk assessment of an asymptomatic individual. Front line health professionals would be expected to interact with and assist asymptomatic individuals through the risk stratification process. In that case, additional knowledge and skills may be needed. Current guidelines and frameworks for genetic competencies of non-specialist health professionals place an emphasis on rare inherited genetic diseases. For common diseases, health professionals do use risk assessment tools but such tools currently do not assess genetic susceptibility of individuals. In this article, we compare the skills and knowledge needed by non-genetic health professionals, if risk-stratified prevention is implemented, with existing competence recommendations from the UK, USA and Europe, in order to assess the gaps in current competences. We found that health professionals would benefit from understanding the contribution of common genetic variations in disease risk, the rationale for a risk-stratified prevention pathway, and the implications of using genomic information in risk-assessment and risk management of asymptomatic individuals for common disease prevention.

## 1. Introduction

Genetic testing will increasingly facilitate a more personalized approach to clinical and preventive healthcare, based on a detailed understanding of underlying pathology and risk. The Collaborative Oncological Gene-environment Study (COGS) [1] identified many new common genetic variants associated with the risk of breast, ovarian and prostate cancer. Modelling studies within COGS showed that risk-stratification based on common susceptibility variants and tailored screening intervention could improve the efficiency of screening programs [2] and improve the benefit to harm ratio including reducing over-diagnosis [3]. Therefore, risk-stratified prevention could replace the “one size fits all” approach to population disease prevention with more tailored interventions. The purpose of risk-stratification for prevention is to recommend preventive interventions for an individual according to an assigned risk stratum. More intensive interventions such as more frequent screening tests or appropriate drugs may be offered to those at higher risk, who have more potential to benefit. Those at lower risk would receive a less intense, potentially less hazardous and less onerous intervention such as a lifestyle intervention.

Currently, comprehensive risk assessment tools that include full family history, lifestyle and personal risk factors as well as genomic information are not yet used for the risk-stratification of asymptomatic people. Using such risk tools, incorporating genomic information, for breast and prostate cancer prevention shows considerable promise, although significant challenges remain [4]. There is a growing body of literature about the potential clinical utility of genomic risk-stratified screening [5,6,7,8]. In modelling studies based on population data, risk-stratified screening based on genetic profiling and age promises to be more efficient with more favorable benefits to harm balance, in relation to the number of people requiring screening, cases detectable and over-diagnosis, when compared to the standard age-based screening [2,3,9]. For example, compared to screening women based on age alone (age 47–73: 10-year absolute risk 2.5%) for breast cancer, a risk-stratified approach for 35–79 year olds could spare 24% of women from screening, at a cost of 3% fewer screen-detectable cases [10]. Similar gains can be seen for prostate cancer screening [10] including reduced overdiagnosis through targeting screening in men with higher polygenic risk [3]. Since Pashayan *et al.* calculated these estimates, further susceptibility alleles have been identified [11], and interest in polygenic risk-stratification has grown [12,13,14]. If all possible susceptibility variants for breast and prostate cancer were known and used in estimating risk, a much larger reduction in the number being screened would be possible, with only a small reduction in cancer-case detection. However, before the implementation of such an approach at the population level, extensive validation of the risk models as well as evidence from population studies, including randomized trials, will be invaluable in understanding issues such as clinical utility, organizational and ethical challenges.

Current research [1,15,16,17,18] is further evaluating the scientific validity, clinical utility, feasibility and acceptability of risk stratification and intervention strategies for those at higher risk. Though a majority of the evaluation is through risk modelling, population-based research is also gaining pace. For example, investigators of the PROCAS (Predicting the Risk of Cancer At Screening) study, claimed to be the “largest study of its kind in the UK which may impact on the whole NHS Breast Cancer Screening Programme” reported that it is feasible to assess and offer risk information and to provide risk-tailored advice to patients undergoing population screening [15,19]. Further empirical work in this study and others such as KARMA (Karolinska Mammography Project for Risk Prediction of Breast Cancer) [16] is ongoing in relation to examining the benefit of improved risk calculations by incorporating single-nucleotide polymorphism (SNP) information and other risk factors within established risk models. Other similar studies include large multicentre European Commission funded projects such as B-CAST (Breast CAncer STratification: understanding the determinants of risk and prognosis of molecular subtypes) and BRIDGES (Breast Cancer Risk after Diagnostic Gene Sequencing) in collaboration with the University of Cambridge [20].

The positive results from modelling studies and the further population based studies that are currently underway, suggest the possibility of inclusion of genetic information for population disease prevention in some shape or form within healthcare systems. This marks the need for policy makers and health professionals to be prepared [21] for this type of change. Such preparation includes identifying and meeting the competence needs of health professionals where the term “competence” delineates the skills, knowledge and attitudes required to undertake a particular task to a nationally recognized level of proficiency. Additional or new competencies may be required by front line health professionals partly because genetic testing for susceptibility and risk stratification in common chronic disease prevention is currently not practiced. In this article we have therefore, performed a timely assessment of the additional competencies that may be needed to implement this type of preventive approach based on genetic risk profiling within the primary care system.

## 2. Experimental

We conducted a competency gap analysis by comparing the knowledge and skills needed by health professionals, not specialized in genetics, for the implementation of stratified prevention with currently recommended proficiencies in established competence frameworks and educational resources.

### 2.1. Multidisciplinary Expert Workshops

Three multidisciplinary international workshops from the COGS project, held between July 2010 and October 2012, enabled us to explore the implications of using genetics in disease prevention [22] including competencies needed by primary care health professionals. The workshop participants had expertise in genetics, epidemiology, social sciences, health economics, primary care, education and screening. We used results from statistical modelling, literature reviews, hexagon modelling [23], group and plenary discussions to build up key strategic themes and to identify issues raised by risk-stratified cancer prevention.

At the workshops, we considered how stratified screening might be delivered. Subsequently we developed proposals in collaboration with some of the workshop participants. As a part of this work, we considered the new competencies that health professionals would need if stratified disease prevention were introduced.

We presented the results at the final workshop in October 2012, along with draft recommendations. These were considered and refined by the participants, and finalized by us after the workshop.

### 2.2. Document Analyses: Current Competence Frameworks and Educational Resources

We assessed the knowledge and skills currently expected in primary care health professionals by reviewing recommended competencies in genetics, genetic risk and risk assessment for non-specialist health professionals. We identified relevant competencies already specified in published frameworks and guidelines publicly available online from the UK, other European countries and North America.

We compared these with the competencies identified from our work in COGS. This enabled us to identify the gaps and additional knowledge and skills required if risk-stratified prevention were to be implemented.

## 3. Results

### 3.1. Competencies Required in Providing Risk-Stratified Cancer Screening

Through the COGS work, three stages of the delivery process of stratified cancer screening were identified: (1) offer of risk-stratified prevention; (2) risk assessment and risk communication; and (3) delivery of personalized intervention [24]. In relation to professional competence, we considered the skills and knowledge that would be required at each of these stages and new aspects that would arise from the integration of genetics within the prevention program, in terms of:

(a) The proposal to include genetic testing as an integral part of the risk assessment tool.
(b) The concept of assigning risk strata to asymptomatic individuals and the offering of preventive intervention only to those at higher risk.

Box 1 summarizes the key components of a general service delivery pathway for prevention based on genetic risk-profiling [24].

Key aspects for which competencies will be required can be grouped under these three stages and are discussed below.

Box 1 **Service delivery pathway for risk-stratified prevention.**

**1. Offer of risk-stratified prevention**

- Invitation of individuals from the general population for risk assessment
- Introducing individuals to risk-stratified prevention including screening, and providing information on its benefits and harms
- Obtaining informed consent and assisting informed decision making

**2. Risk assessment and risk communication**

- Genetic testing and assessment of non-genetic factors
- Using a risk calculator or risk tool to integrate genetic and non-genetic information in order to get a risk score and assign a risk category
- Communication of risk assessment results
- Addressing concerns and anticipated psychological impact of test-results

**3. Delivery of personalized intervention, e.g., screening**

- Tailoring of interventions according to risk category (or no intervention/general advice to those at low risk)

#### 3.1.1. Offer of Risk-Stratified Prevention

##### 3.1.1.1. Invitation for Risk Assessment

Health professionals would need to understand the eligibility criteria and the call/recall process for risk assessment. They would need to understand and be able to explain the basis for identifying the population who would be invited to the stratified prevention and screening program, in particular the age of entry to the program, if different from standard screening programs.

##### 3.1.1.2. Explaining Risk-Stratified Prevention and Assisting in Informed Decision Making

Health professionals would need to understand and be able to explain the underlying rationale for a risk-stratified prevention program, including the tailoring of prevention options according to risk stratum. Health professionals would need to know about and be able to explain the available prevention options, such as screening and behavioral intervention, including the possible harms of false positive and false negative results, psychosocial, privacy as well as discrimination aspects [25,26,27]. The individual undergoing risk assessment would also need to be informed about the recording, retaining, storing and sharing of their personal information including genetic data as well as the protections in place for ensuring that their data is not disclosed inappropriately [28].

##### 3.1.1.3. Understanding the Contribution of the Genomic Information

In order to understand how genetic data contributes to the risk-assessment, health professionals need to have a broad understanding of the assay used to generate the genetic variant information, the inherent advantages and disadvantages associated with different methods, and be able to communicate these in a clear and concise manner. In the short term, targeted methods such as SNP genotyping are most likely to be adopted, but in the future, the use of next generation sequencing methods such as candidate gene panels, or ultimately whole exome or genome sequencing increase the potential for incidental/unsolicited findings to be generated which could have implications for the patient [29].

#### 3.1.2. Risk Assessment and Risk Communication

Clinical professionals are expected to be able to use a risk tool and to interpret and apply the risk scores of the individual.

##### 3.1.2.1. Integration of Risk Factors and Risk-Assessment

Individual risk assessment may involve taking relevant personal and family history, lifestyle information and environmental exposures, making appropriate anthropometric and biometric measurements and obtaining relevant biomarker measurements and using a valid risk tool for risk profiling. Non-specialist professionals need to be aware of the range, relevance and weight of key genetic variants and any other biomarkers in informing individuals’ disease risk and their contribution in the risk-assessment tool. Professionals must be knowledgeable about the range of genetic susceptibility variants relevant to the particular cancer including variants with low (e.g., SNPs), intermediate (e.g., *CHEK2* mutations) or higher predictive value (such as *BRCA1/2* mutations) and the significance that these may have for the individual and their family members. In addition to the individual risk assessment, health professionals should also be able to distinguish individuals at a very high risk with highly penetrant alleles such as *BRCA1/2* (if they are included in the risk assessment) and refer them appropriately. Overall, they must be able to explain individual risk based on the different risk factors.

##### 3.1.2.2. Communication of Results

Health professionals will need knowledge of the nature and determinants of cancer risk, have an understanding about the complexities of individual perspectives of risk, and must be effective communicators. They have to respond appropriately to the queries of the individual which may range from questions about the use of risk assessment to predict disease risk, the use of genetic/genomic, biometric and environmental/lifestyle information, the accuracy of risk prediction and the possibility of incidental findings.

They will also be expected to communicate effectively if patients are found to have highly penetrant alleles and when discussing discordance or concordance in results of family history assessment with results of genetic tests for common variants. Health professionals must be trained to communicate risk scores effectively in a way that is jargon free, easily understood, and takes into account the individual’s background, values and beliefs [30], and which supports patients in their decision making about prevention options including screening. Clear communication and reassurance would be particularly needed for those individuals, who were previously eligible for screening, but are found to be at low risk and are not offered intervention under a risk-based screening program [31]. Younger women at higher risk and eligible for screening under a new system, but ineligible under previous programs, would also need to be counselled appropriately. Communication should also include information about the possibility and degree of false positive and false negative risk assessment results as well as relevant future implications. Whilst some of these communication skills are not necessarily unique to risk-assessment using genomic information, the distinct element is in relation to using genetic information in risk assessment of individuals without family history and in chronic disease prevention. Since genomic knowledge in the context of risk-stratified prevention or screening strategies is not currently being used for this purpose, the relevant risk-communication needs may need further evaluation.

##### 3.1.2.3. Addressing Concerns and Anticipated (Psychological) Impact of Test-Results

Specific anxieties related to risk stratified prevention may need to be addressed. Individuals may have concerns about their genetic information being used in assessing disease risk. For example, they may have questions about the implications of the risk assessment result for family members, including children, and in relation to third parties such as insurers and employers. Furthermore, they may need information about the consequences of new genetic findings/variants. Health professionals should also be aware that risk assessment test results whether positive or negative, may influence individuals’ perception of risk [32,33], cause short term psychological effects on individuals [34,35] such as anxiety and may or may not influence their behavior including a change in lifestyle [36,37].

#### 3.1.3. Delivery of Personalized Intervention

Health professionals need to be able to use the individual risk score from a risk assessment test to offer an appropriate intervention, for example more frequent screening for those at higher risk and no intervention or general advice to those at low risk. If a stratified screening intervention is offered, they should understand the reasons behind this choice at a policy level and be able to explain it at the level of the individual patient.

### 3.2. Current Recommendations for Competencies in Genetics and Risk-Assessment

#### 3.2.1. Genetic Competencies

Genetic competence frameworks are formally developed in collaboration with genetic specialists, healthcare professionals, patient groups and educators in order to ensure their appropriate delivery. One of their main purposes is to provide clear goals for structured learning and to define learning outcomes in the development of training and learning programs. Examples of frameworks relevant to stratified prevention and those related to genetics, genetic risk and risk assessment in general are listed in Box 2 and include examples from Europe (EuroGentest) [38], USA, (National Coalition for Health Professional Education in Genetics) [39], and the UK (Royal College of General Practitioners) [40].

Box 2 **Examples of current competence frameworks in relation to genetics and risk assessment.**

GeneticsCore Competences in Genetics for Health Professionals in Europe [38]Framework for development of physician competencies in genomic medicine, the Intersociety Coordinating Committee for Physician Education in Genomics (ISCC), USA, 2014 [41]Core Competencies in Genetics for Health Professionals (2007), NCHPEG, USA [39]Genetics in Primary Care, Royal College of General Practitioners Curriculum 2010 (Revised 2014), UK [40]Genetic riskGTC6 Assessing a genetic risk, UK [42]Risk assessmentNHS Health Check competence framework, UK [43]

Though there are significant differences in practical delivery of professional education and how health practice is regulated across developed countries [44], frameworks for core competencies in genetics do not vary substantially. The common themes in most genetic competence frameworks for non-genetic specialist health professionals are those necessary to assess, identify, manage and support individuals with inherited genetic disorders or conditions [38,39,45,46,47,48]. Box 3 highlights core genetic competencies that are common in many frameworks for non-genetic specialists; and detailed guidance provided for various non-specialist health professionals is tabulated (Table 1). However, we recognize that these competencies might differ slightly to take into account various professional roles (e.g., that of a practice nurse compared to a general practitioner).

The recently revised Royal College of General Practitioners’ guideline for genetic competence in UK primary care highlights the importance of knowledge about genetic susceptibility in common conditions and the use of genetic information in stratified medicine. It also includes recommendations for primary care professionals to be able to use online risk assessment tools as they become available [40]. Recent progress also includes the creation of a framework, by a working group in the USA, for the development of genomic competencies guidelines in medical disciplines [41].

Box 3 **Common themes in present primary care genetics competence frameworks.**

1. Knowledge of genetics, signs, symptoms in genetic disorders
2. Identify individuals with or at risk of a genetic condition
3. Assess family history for predisposition to disease
4. Communicate genetics information for informed decision
5. Manage patients with genetic conditions
6. Obtain specialist help on inherited conditions
7. Understand relevant ethical, social and legal issues and offer appropriate psychological and social support

**Table 1:** Current recommendations: Core competencies in genetics for health professionals (non-specialists in genetics).

	Common Competence Themes	Core Competences in Genetics for Health Professionals in Europe [38]	Core Competencies For All Health Professionals (2007), NCHPEG, USA [39]	Genetics in Primary Care, Royal College of General Practitioners Curriculum 2010 (Revised 2014), UK [40]
1.	Knowledge of genetics, signs, symptoms in genetic disorders	Demonstrate an understanding of heterogeneity in genetic diseases and the principles of assessing genetic risk.	Understand basic human genetics terminology. Understand the basic patterns of biological inheritance and variation, both within families and within populations. Understand how identification of disease-associated genetic variations facilitates development of prevention, diagnosis, and treatment options. Understand the interaction of genetic, environmental, and behavioural factors in predisposition to disease, onset of disease, response to treatment, and maintenance of health. Use information technology to obtain credible, current information about genetics.	Be aware that variations in the human genome may have no effect, may lead to a predisposition to common diseases (such as coronary artery disease or cancer), or may result in serious conditions in a significant minority of your practice. Demonstrate an awareness that it is not always possible to determine the cause of a condition (e.g., a learning disability) that may be genetic in origin, nor the mutation responsible for a genetic condition. Demonstrate an awareness of the genetic aspects of antenatal and newborn screening programmes (e.g., Down’s syndrome, cystic fibrosis, sickle cell and thalassaemia) and know their indications, uses and limitations, and from where to obtain information. Demonstrate an awareness that genetics is a rapidly evolving area, to keep up to date with clinical advances and their implications on ethical debate and service planning; to be up to date about how genomic information can contribute to risk factors in common conditions and the personalisation of management through stratified use of medicines.
2.	Identify individuals with or at risk of a genetic condition	Demonstrate awareness that the make-up of the local population may affect the prevalence of genetic conditions and attitudes towards genetic disease. Demonstrate awareness that reassurance is the appropriate action for patients who have been assessed as being at population risk. Advise patients about relevant screening programs as appropriate.	Understand the difference between clinical diagnosis of disease and identification of genetic predisposition to disease (genetic variation is not strictly correlated with disease manifestation). Gather genetic family history information, including at minimum a three-generation history.	Describe how to identify patients with, or at risk of, a genetic condition through considering the family history and applying knowledge of inheritance patterns, or patients with diagnoses known to have a genetic cause.
3.	Genetic risk and risk assessment (particularly in common diseases)	Not mentioned.	Not mentioned.	Demonstrate an awareness of the heterogeneity in genetic diseases and understand the principles of assessing genetic risk, e.g., principles of risk estimates for family members of patients with Mendelian diseases; principles of recurrence risks for simple chromosome anomalies, e.g., trisomies; the use of information from susceptibility loci in common complex conditions; the ability to use online risk assessment tools as they become available.
4.	Family history in assessing predisposition to disease	Know how to take and interpret a family history Understand the relevant inheritance patterns and mechanisms by which genetic disease may occur Use a family history and knowledge of inheritance patterns to identify those patients in the practice population with, or at risk of, a genetic condition.	Understand the importance of family history (minimum three generations) in assessing predisposition to disease.	Demonstrate an appreciation of the importance of identifying families with autosomal dominant conditions such as familial hypercholesterolaemia and polycystic kidney disease to ensure that affected family members receive appropriate treatment, and the importance of offering carrier testing for families with autosomal recessive conditions such as sickle cell, thalassaemia or cystic fibrosis. Demonstrate an awareness of the different implications for other family members depending on the genetic cause of a condition (autosomal dominant and recessive and X-linked single-gene inheritance; de novo and inherited chromosomal anomalies; mitochondrial inheritance and somatic mutation). Be able to take and interpret a family history. This involves: Knowledge of relevant questions; knowledge of basic inheritance patterns (autosomal dominant and recessive, X-linked, mitochondrial, multifactorial); understanding that while some genetic conditions always present with the same signs and symptoms, others can show variability between family members, particularly some autosomal dominant conditions (such as neurofibromatosis type 1).
5.	Communicate genetics information for informed decision	Demonstrate awareness that genetic information impacts not only on the patient but also on their immediate and extended family. Use appropriate communication skills and demonstrate awareness of the need for confidentiality and a non-directive approach. Consider the patient’s cultural and religious background and beliefs concerning inheritance when providing care for people with, or at risk of, genetic conditions.	Understand the various factors that influence the client’s ability to use genetic information and services, for example, ethnicity, culture, related health beliefs, ability to pay, and health literacy. Understand the resources available to assist clients seeking genetic information or services, including the types of genetic professional available and their diverse responsibilities. Explain effectively the reasons for and benefits of genetic services. Assure that the informed-consent process for genetic testing includes appropriate information about the potential risks, benefits, and limitations of the test in question.	Demonstrate appropriate skills to communicate information to patients about genetics in a comprehensible way with particular awareness of the need: for confidentiality when information received from or about one individual can be used in a predictive manner for another family member in the same practice to remain non-directive and non-judgemental Demonstrate an awareness that consultations involving the giving of genetic information and discussion may require more time.
6.	Manage patients with genetic conditions	Demonstrate an awareness of the different uses of genetic tests (diagnostic, predictive, carrier testing) and their limitations. Demonstrate an awareness of the need to ensure that systems are in place to follow-up patients who have, or are at risk of, a genetic condition and have chosen to undergo regular surveillance.	Assumed*	Demonstrate comprehensive management for those patients with, or at risk of, genetic conditions through coordination of care with other primary care professionals, geneticists and other appropriate specialists. This is particularly important because genetic conditions are often multisystem disorders. Describe the reproductive options available to those with a known genetic condition (including: having no children; adoption; gamete donation; prenatal diagnosis).
		Demonstrate an awareness that preventative measures exist for some genetic conditions and advise patients of these. Describe the reproductive options available to those with a known genetic condition, (including: having no children; adoption; gamete donation; pre-natal diagnosis). Demonstrate an awareness of antenatal and other screening programmes for genetic conditions and know where to obtain information on these programmes for themselves and for patients. Understand and use the national guidelines that influence healthcare provision for those with genetic conditions.	Assumed*	Describe how to access guidelines for managing patients with genetic conditions (such as familial hypercholesterolaemia or sickle cell disease). Demonstrate an awareness of the need to ensure that systems are in place to follow up patients who have, or are at risk of, a genetic condition and have chosen to undergo regular surveillance (for example: imaging for breast cancer and for adult polycystic kidney disease; or endoscopy for colon cancer). Demonstrate an awareness that preventative measures or targeted treatments exist for some genetic conditions (for example: lifestyle interventions; mastectomy and/or oophorectomy for BRCA1/2 mutation carriers; colectomy for adenomatous polyposis coli mutation carriers; statin use for familial hypercholesterolaemia; venesection for haemochromatosis; losartan for patients with Marfan syndrome). Describe the support services available for those with a genetic condition (e.g., Contact a family).
7.	Obtain specialist help on inherited conditions	Understand the organisation of genetics services in his or her region or country. Understand the limits of his or her own genetics expertise and know when and where to seek advice. Make appropriate referrals to genetics services conditions. Demonstrate awareness that, because genetic conditions are often multi-system disorders, comprehensive patient management is likely to involve liaison with other healthcare professionals.	Understand one's professional role in the referral to or provision of genetics services, and in follow-up for those services. Identify and refer clients who might benefit from genetic services or from consultation with other professionals for management of issues related to a genetic diagnosis. Seek coordination and collaboration with and interdisciplinary team of health professionals.	Describe local and national referral guidelines (for instance, for a family history of breast or colon cancer). Describe the organisation of genetics services and how to make appropriate referrals.
8.	Understand relevant ethical, social and legal issues and offer appropriate psychological and social support	Demonstrate awareness that genetic information may have ethical, legal and social implications. Demonstrate an awareness of the emotional impact of a genetic diagnosis on a patient and their family. Discus with the patient the range of support services available, both professional and lay. Demonstrate an awareness of the importance of the social and psychological impact of a genetic condition on the patient and their family, dependants and employer.	Understand the potential physical and/or psychosocial benefits, limitations, and risks of genetic information for individuals, family members, and communities. Understand the ethical, legal, and social issues related to genetic testing and recording of genetic information (e.g., privacy, the potential for genetic discrimination in health insurance and employment). Appreciate the sensitivity of genetic information and the need for privacy and confidentiality.	Demonstrate an awareness of the potential emotional, psychological and social impacts of a genetic diagnosis on a patient and his or her family, particularly associated with guilt about ‘passing on’ a condition. Demonstrate an awareness of the ethical issues that may arise, including confidentiality and non-disclosure of genetic information within families; genetic testing in children; the 'right not to know' and exercising care in the use of information (for instance in access to insurance or employment issues). Demonstrate an awareness of the different uses of genetic tests (diagnostic, predictive, carrier testing), their limitations and ethical considerations (for instance associated with testing in children and with presymptomatic testing). Demonstrate an awareness that the makeup of the local population may affect the prevalence of genetic conditions and attitudes towards genetic disease. Demonstrate an awareness that a patient’s cultural and religious background and beliefs concerning inheritance and genetics are important to consider in providing care for people and families with, or at risk of, genetic conditions. An example of a belief concerning inheritance is that a particular genetic disease in a family is linked with a particular physical appearance.

* It is assumed that these competences are thought to already exist or are expected. These may not have been directly mentioned in the framework.

#### 3.2.2. Competence in Formal Risk-Assessment

Primary care health professionals in many countries use validated risk calculators in clinical preventive practice. Risk calculators are essentially mathematical algorithms that generate a score or risk category based on patient details including personal information such as age, sex, personal and family history, lifestyle, physiological measurements and biomarkers.

Risk assessment is currently practiced for preventing common diseases such as cardiovascular disease (CVD) [49,50,51], diabetes [52], breast cancer [53], and fracture [54]. In many developed countries, national and international risk assessment tools are available for assessing common disease risk [55,56,57] and there are guidelines for managing disease risk [58,59]. For example, as a part of the National Health Service (NHS) Health Check in the UK, individuals are assessed for the risk of developing heart disease, stroke, diabetes, kidney disease and some forms of dementia through regular risk assessments. A national document, the *NHS Health Check Competence Framework,* specifies that health professionals carrying out an NHS health check should be able to conduct targeted health screening programs, use and share confidential personal data in a fair and lawful way, understand the eligibility and the recall process, obtain informed consent, be competent in risk assessment, and be able to interpret and communicate risk score results as well as give health advice appropriately [60]. Competences recommended by the NHS for conducting a CVD risk assessment are listed in Box 4.

Box 4 Current key competencies recommended for carrying out risk assessment for cardiovascular disease * (Source: CVD EF3, NHS Skills for Health [61]).

**Performance Criteria****Knowledge and Understanding**Need to explain
The role of the health professionalThe information to be obtained, stored and sharedWhat is involved in the assessmentGeneric work related knowledgeHow to ask questions, listen and summarizeHow to ask questions, listen and summarizeHow to record, store and share informationMaintain confidentialityRespect individuals’ privacy, dignity, wishes and beliefsGeneric healthcare knowledge
Anatomy, physiology, biochemistryPrinciples and methods of informed consentClinical examinationAnthropometric and biometric measurementsMinimize discomfort, encourage participation Obtain informed consent**Use appropriate tools and methods****Specialist healthcare knowledge**
**Nature and determinants of the (cardiovascular) disease including lifestyle****Interpreting indicators or risk and symptoms****Interpreting results of tests and measurements****Calculating individuals risk-level using validated tool****Take family history and lifestyle information****Note individual symptoms****Assess other conditions****Calculate level of risk****Refer if needed**
Context specific knowledge
Acquire relevant local knowledge for health contextUnderstand own role, responsibility and accountability

* Competencies particularly relevant to risk-assessment for stratified prevention are highlighted.

### 3.3. The Competence Gap for a Genetic Risk-Stratified Prevention Program

We compared the primary care competencies necessary for implementing a risk-stratified prevention program with those currently recommended within the competence frameworks of various countries. The area least well covered by existing frameworks was knowledge and skills about the use of genetic susceptibility variants in risk assessment. Only the UK Framework indicates the need to know that common susceptibility variants may lead to predisposition to common diseases. The UK guideline, however, does not refer to using genetic variant information in future risk tools, and the other frameworks do not recommend knowledge and skills in relation to genetic risk and risk assessment, particularly that in common diseases. Nor do they cover tailored interventions for those at higher risk or communicating the harms and benefits of risk-stratification.

From our assessment, it appears that there are certain gaps in the current competences of front-line health professionals in relation to the knowledge and skills surrounding the use of genomic information in the context of risk assessment tools for the asymptomatic population. The additional competencies that may, therefore, supplement the current recommendations from Table 1 are listed in Table 2.

## 4. Discussion

In this paper, we have performed an assessment of the additional competencies that may be needed in preparation of the use of genomic profiling in risk assessments within the primary care system. This is based on the assumption that sufficient evidence, once available, may support the implementation of population risk-stratified prevention in the future. We compared the current genetic and risk assessment competence guidelines from some developed nations with competencies required for risk-stratified prevention incorporating genetic information. We found that a majority of the publicly available competence frameworks in genetics for frontline health professionals cover the basic requirements for understanding the genetic basis of diseases and management of patients, principally for those with inherited genetic conditions. The UK also provides competence frameworks for risk assessment for some common diseases. We identified that additional competencies were needed, primarily about knowledge of the role of common susceptibility variant information in disease prevention, about the rationale and pathway for a risk-stratified prevention program incorporating genomic information, and about the implications of using genomic information in risk assessment leading to stratified prevention in healthy individuals (Table 2).

**Table 2:** Additional competencies required for risk-stratified prevention based on genetic profiling.

	Competence Themes	Additional Competencies Needed if Genomic Profiling Included in Risk Stratification
1	Knowledge of genetics, signs, symptoms in genetic disorders	Be aware of the extent and weight of contribution of common genetic variants and other determinants in contributing to disease risk and of their relevance in a risk assessment tool
2	Identify individuals with or at risk of a genetic condition	Competence already recommended in established frameworks
3	Genetic risk and risk assessment (particularly in common diseases)	Understand the rationale and pathway of the risk-stratified prevention programs incorporating genomic information
4	Family history in assessing predisposition to disease	Be able to explain any discordance in the relationship between the results of patients’ genetic test for common variants and their family history assessment
5	Communicate relevant genetic information to enable informed decision making	Be aware of the specific harms and benefits arising from incorporating genetics in risk assessment tools. Understand and provide information on the range and relevance of key genetic variants included in the test including the difference in risk contribution by high penetrant (e.g., variants within *BRCA 1/2*) and low penetrant alleles (e.g. single nucleotide polymorphisms)
6	Manage patients with genetic conditions	Health professionals are assumed to be competent in tailoring prevention interventions according to risk category
7	Obtain specialist help on inherited conditions	Competence already recommended in established frameworks
8	Understand relevant ethical, social and legal issues and offer appropriate psychological and social support	Respond to concerns about implications of the genetic component of a risk assessment result for family members Explain, as appropriate, how the information obtained, including genetic data, may be shared with others including researchers, and, as appropriate, with commercial organisations, insurers or employers, and respond to specific concerns Explain how the information obtained, including genetic data, will be used and stored, and be able to respond to specific ethical, legal and social concerns of the patient

The extent of additional medical education or professional training required, in relation to the new competencies, does not appear to be extensive and may be resolved by the development of specific educational resources or their integration into existing educational and training programs. Consequently, assuming that front line health professionals are currently receiving the education and training recommended in the national competence guidelines, it is unlikely that there will be any significant rise in training or infrastructure costs in relation to additional competence development for risk-stratified prevention strategies. We nevertheless suggest that relevant empirical studies include an assessment of the extent of these associated costs. In our evaluation, we have assumed that frontline health professionals are competent in genetics and in conducting risk-assessments in accordance with recommendations in formal guidelines. However, there is evidence that health professionals in primary care have some deficiencies in the knowledge and skills required to effectively deliver current genetic services [62,63,64,65]. We therefore recommend that initially a formal education and training needs assessment exercise is conducted to determine current gaps in genetic competencies. Appropriate empirical studies will also be invaluable in our understanding about gaps and future competence needs. Existing gaps as well as those related to delivery of prevention based on genetic profiling can then be addressed through appropriate programs. The newer aspects of competence requirements in relation to risk-stratified prevention could then be embedded into the curricula of pre- and post-registration programs and Continuing Professional Development (CPD) programs for health professionals.

Awareness of the importance of genetic and genomics education and training is growing at present including that in the UK [66,67] and the USA [41]. Health Education England, the body responsible for the education, training and personal development of NHS staff has taken the lead for preparing the NHS workforce for genomics in order to support the 100,000 Genomes Project of Genomics England [68]. Their key objectives include embedding genomics into education and training for the prospective workforce and commissioning additional training schemes, fellowships and CPD programs. Interactive peer resource workshops and online resources will be made available to ensure a more integrated approach to genomics training. We suggest that such programs be extended to include material about the genetic basis of common diseases as well as about the prospective use of genetic information and risk assessment tools to calculate the risk of an asymptomatic individual for common diseases such as breast and prostate cancer.

## 5. Conclusions

Risk stratification for the prevention of common chronic disease would involve genetic profiling for risk assessment in asymptomatic people. This marks a move from the current ‘one size fits all’ programs and adds refinement to programs aimed at identifying only high risk individuals, by stratifying the whole population into a number of risk categories with appropriate interventions offered across the entire spectrum. If such programs are to be implemented effectively, professionals will need to understand and explain their rationale and deal responsibly with concerns that arise. We have indicated that various formal competence frameworks targeted at primary healthcare professionals already cover the majority of the knowledge and skills required for implementation of stratified prevention. What is additionally required is a greater emphasis on the knowledge related to the use of common susceptibility variant information in risk assessment tools and the concept of tailored prevention in asymptomatic individuals. As a first stage, we recommend that a more formal educational needs assessment should be undertaken to establish the current baseline, the precise knowledge and skills required by the various health professionals and to establish the most effective ways of supporting the necessary learning related to issues around genetic service delivery. Appropriate additions to the educational and training curriculum can then be made to address the current gaps, as well as the additional competencies needed, in relation to the prospective use of genomic information in the prevention of certain common diseases. Such preparation of health professionals for the implementation of new genomic knowledge and technologies into their everyday practice will be indispensable if the full potential of the major international investment in genomics is to be realized.
